# Life Course Stressors and Functional Limitations in Later Life Among White, Black, and Hispanic Adults

**DOI:** 10.1093/geroni/igab046.814

**Published:** 2021-12-17

**Authors:** Madison Sauerteig, Kenneth Ferraro, Shawn Bauldry

**Affiliations:** 1 Purdue University, West Lafayette, Indiana, United States; 2 Purdue University, Purdue University, Indiana, United States

## Abstract

Although striking racial and ethnic disparities in health are manifest during later life, they may be rooted in early-life exposures. Drawing from cumulative inequality theory, we investigate whether experiencing life course stressors increases the risk of later-life functional limitations and whether this relationship differs by race and ethnicity. This study utilizes longitudinal data from the Health and Retirement Study to test whether six indicators of child stressors and eleven indicators of adult stressors predict trajectories of the onset and severity of functional limitations in later life among a diverse sample of adults. We find that child and adult stressors are associated with earlier onset and greater severity of functional limitations during later life. Mediation analyses reveal the indirect influence of child stressors via adult stressors on onset and severity of functional limitations; however, the indirect effects are slightly stronger for Black and Hispanic adults than their White counterparts (i.e., moderated mediation). In sum, child stressors, in and of themselves, do not increase functional limitations among Black and Hispanic people but are associated with greater adult stress exposure, leading to more functional limitations in later life. Disparities in functional limitations are also partly due to lower education and less wealth among Black and Hispanic adults.

